# The Calabar Bean

**Published:** 1864-10

**Authors:** 


### THE CALABAR BEAN.

### PHYSOSTIGMA VENENOSUM.

The want of a remedy has long been felt which would more thoroughly act as an antagonistic to atropine and other mydriatics than opium or ergotine, in producing contraction of the pupil. This agent has been found in Calabar bean. The drug was first mentioned by Christison in 1855; but only its action when taken internally had been examined. Professor R. Fraser, also of Edinburgh, drew attention eight years afterwards to the myotic qualities of the remedy. And we copy the following from his remarks:

The plant from which the beans are obtained is a runner, growing wild in West Africa, and is gathered by the negroes, who employ it as an ordeal for criminals. The population of Calabar is estimated at one hundred thousand, and of these upwards of 120 are reckoned annually to be sacrificed by this poison.

These so called beans average rather more than an inch in length, and are irregularly reniform, having the appearance of a somewhat flattened fusiform body bent on one of its edges.

As obtained from Calabar, the beans have a grey color, and are encrusted with earthy matter; this is readily removed by washing. and a somewhat shining integument is exposed of various shades of brown, ranging from a light coffee to an almost perfect black.

While the other parts of the plant are indifferent, as it seems, to the animal organism, the beans have strongly poisonous qualities. According to the missionaries, those who have eaten them, first feel a violent thirst; afterwards, the poisoned individual cannot swallow; mucus flows from the mouth; convulsions; and also cramps in the back, come on, etc. During all this, the patient is conscious of everything, and even the language remains up to shortly before death, which may ensue within half an hour. Sometimes vomiting occurs, after which the heat diminishes; and, except headache, all other symptoms disappear.

Small doses (up to 12 grains) with which Christison made his experiments, soon caused an increasing pain in the epigastrium, with retching, a sentiment of dyspnœa, cramps in the muscles of the breast, vertigo and weakness in the limbs, great secretion of saliva, and irregular, slow motion of the heart, so that in one case the pulse made only 20 beats.

Applied locally to a rabbit, the alcoholic extract produced loss of contractility. And when the intestines were painted over with a solution, they ceased to move.

In order to examine the local action of the remedy on the eye, Robertson prepared an extract from the pulverized bean, which he dissolved in alcohol in three different concentrations. The weakest solution was produced by extracting 30 grains of the bean by alcohol, evaporating to desiccation and dissolving the rest in one drachm of water. Thus a dirty light red-brown fluid was obtained. By further extracting and evaporating, a four times and an eight times stronger extract were obtained. After R. had examined his eyes, and found that his pupils were 2 lines in diameter each, and with each eye Jager's No. 1 was read at 5 inches distance, he put a drop of the weakest solution in his left eye, which did not produce any more irritation than a drop of water. After 10 minutes, objects at a distance of one foot became indistinct; at the same time all objects seemed larger and nearer. There existed also a sensation of tension in the eye, as if very minute objects had been assiduously looked at. Both pupils were yet equal in size. After twenty minutes the left pupil had only a diameter of 1 line; objects further distant than 9 inches appeared dim; every thing looked at seemed larger and nearer. The right eye was normal. After 30 minutes the left pupil was only $\frac{3}{8}$, the right one $2\frac{1}{2}$ lines in diameter. The far-point of the left eye 8 inches. After 50 minutes the left pupil was $\frac{1}{2}$ and the right one 2 lines; a sensation of pressure and fatigue became manifest, when the subject of the experiment attempted to read; objects at 10 yards distance were recognized with difficulty. After 6 hours the left pupil was 1 line, the right one $1\frac{1}{2}$ lines; both reacted on light. After 12 hours the left pupil was $1\frac{1}{2}$, the right one $2\frac{1}{2}$ lines in diameter. The following morning both pupils showed yet a slight difference and the left eye was somewhat weak.

Bowman, who has also experimented on his own eye, as stated by Wells, says that after 5 minutes he felt a strong tension in the neighborhood of the ciliary body, as if something crept about there. After 10 minutes this sensation had yet increased, and he felt also some lancinating pain. After 15 minutes the near-point was at the left side $6\frac{3}{4}$ inches, while in the right eye, to which nothing had been applied, it was removed to a distance of 15 inches. The far-point seemed equally distant in both eyes. After 20 minutes No. XVII Jager was seen at 15 feet, but the letters oscillated; they disappeared and returned alternately. The left pupil was then contracted to the size of the head of a pin, remained in this state for 18 hours, and in the course of three days again became normal. With this dilatation the reaction of the pupil on light again became noticeable on both eyes. Twenty-five minutes after the application there existed astigmatism: the vertical staffs of a window appeared perfectly distinct at a distance of from 6 to 10 feet, while the horizontal ones seemed dim and angular. This was remedied by a concave cylindrical glass of 14 inches focus. With a cylindrical glass of 50 inches focus distant objects appeared palpably smaller. This astigmatic state was yet found 18 hours after the application.

De Graefe has tested the new myotic on 9 healthy persons. The average time for the setting in of contraction was 14 minutes with the weak, 12 minutes with the strong solution; the duration of contraction with the former 2, with the latter 3 days; the maximum of contraction lasted from 6 to 18 hours. The altered state of refraction, *i. e.* the cramp of the muscle of accommodation and the approach of the near-point, lasted much less in Graefe's experiments: it reached its height in 10 minutes, and remained there but from 10 to 20 minutes. The apparent increase in the size of objects and change of illumination were also observed; the acuteness of vision was reduced from 1 to $\frac{2}{3}$. Ophthalmoscopically there appeared no change of circulation. In a patient who had no iris, but good vision, the action on the ciliary muscle was also manifest. Experiments on birds showed the action of the drug on the pupil of these animals to be very brief; on amphibia and fishes the remedy remained without influence. De Graefe also satisfied himself that atropine is a much more powerful irritant in an opposite sense than the Calabar bean. The latter is not capable of contracting the pupil after it has just been dilated by atropine; the action of the latter also again appeared, when in an atropinized eye the Calabar bean had for a short time produced a medium degree of contraction. When the pupil had first been contracted, atropine always acted, but somewhat slowly. The remedy acted also on the iris, when it was abnormal but not totally atropic, in glaucoma and in a case of fistula of the cornea.

From all hitherto published experiments, it results that the Calabar bean first produces a subjective sensation of tension in the ciliary body, which may be recognized also by the determination of the near-point and the range of accommodation; that it also causes contraction of the pupil; that the contraction reaches its height in the course of an hour; that the iris loses during that period its contractibility; and that the dilatation to the normal size from this contracted state requires less time than the contraction of the pupil, when dilated by atropine (the latter circumstance probably depends on our incapacity up to the present time to extract entirely the active principles of the bean.) Simultaneously with the tension of the ciliary body occur the symptoms of myopia with a small range of accommodation, and of astigmatism. The remedy, therefore, acts by producing a cramp, by irritation of the ciliary branches of the oculo-motorius, in the ciliary muscle and sphincter of the pupil; it does not paralyze the dilatator pupillæ, as otherwise it could not produce complete contraction in a previously dilated pupil. It is, consequently, in so far an antagonist of atropine as the latter irritates the dilatator of the pupil.

Therapeutically the remedy will find the following applications: 1. Perhaps in retinitis, with great sensibility to light, in order to moderate the admission of light. 2. In mydriasis, consecutive in some cases to debilitating diseases (typhus, diphtheritis, etc.), and to injuries. 3. In ulcers at the margin of the cornea, in order to avoid incarceration of the sphincter of the pupil after perforation. 4. In artificial mydriasis, in order to do away with the dazzling, which is very disagreeable to the patients after having been examined by the ophthalmoscope, particularly if they have but one eye. [To these indications may be added the following: 5. In those corneal opacities with a transparent centre, which produce, when the pupil has its normal size, dazzling by diffusion of light. 6. In similar circumscript opacities of the crystalline body, situated near the centre of the latter, and in dislocations thereof. 7. In abnormal mobility of the lens, with a tendency to fall into the anterior chamber. 8. For the discovery of simulated amaurosis, the pupil being dilated with atropine. 9. In wounds of the cornea and sclerotic with a recent prolapsus of the pheripheric part of the iris. 10. Perhaps in diseases of the ciliary body.]

E. Hart recommends a solution of the extract of such a concentration that one drop shall contain the extractive matter of three grains of the crude drug. He furthermore says that the alcoholic extract is soluble in glycerine, and that this solution is more durable than the watery one, which after a few days becomes decomposed.—GEISSLER. *American Jour. Ophthalmology.*

